# Screening of *WT1* mutations in exon 8 and 9 in children with steroid resistant nephrotic syndrome from a single centre and establishment of a rapid screening assay using high-resolution melting analysis in a clinical setting

**DOI:** 10.1186/s12881-016-0362-7

**Published:** 2017-01-10

**Authors:** Annes Siji, Varsha Chhotusing Pardeshi, Shilpa Ravindran, Ambily Vasudevan, Anil Vasudevan

**Affiliations:** 1 Division of Molecular Medicine, St. John’s Research Institute, Bangalore, India; 2 Department of Pediatric Nephrology, St. John’s Medical College Hospital, Bangalore, 560034 India

**Keywords:** Children, *WT1*, Steroid resistant, High resolution melt, Mutation

## Abstract

**Background:**

Mutations in Wilm’s tumor 1 (*WT1*) gene is one of the commonly reported genetic mutations in children with steroid resistant nephrotic syndrome (SRNS). We report the results of direct sequencing of exons 8 and 9 of *WT1* gene in 100 children with SRNS from a single centre. We standardized and validated High Resolution Melt (HRM) as a rapid and cost effective screening step to identify individuals with normal sequence and distinguish it from those with a potential mutation. Since only mutation positive samples identified by HRM will be further processed for sequencing it will help in reducing the sequencing burden and speed up the screening process.

**Methods:**

One hundred SRNS children were screened for *WT1* mutations in Exon 8 and 9 using Sanger sequencing. HRM assay was standardized and validated by performing analysis for exon 8 and 9 on 3 healthy control and 5 abnormal variants created by site directed mutagenesis and verified by sequencing. To further test the clinical applicability of the assay, we screened additional 91 samples for HRM testing and performed a blinded assessment.

**Results:**

*WT1* mutations were not observed in the cohort of children with SRNS. The results of HRM analysis were concordant with the sequencing results.

**Conclusion:**

The *WT1* gene mutations were not observed in the SRNS cohort indicating it has a low prevalence. We propose applying this simple, rapid and cost effective assay using HRM technique as the first step for screening the *WT1* gene hot spot region in a clinical setting.

**Electronic supplementary material:**

The online version of this article (doi:10.1186/s12881-016-0362-7) contains supplementary material, which is available to authorized users.

## Background

Steroid resistant nephrotic syndrome (SRNS) remains one of the most common intractable causes of end-stage renal disease (ESRD) in children with 50–70% reaching ESRD within 5–10 years of diagnosis [1, 2]. Discovery of pathogenic variants in at least 40 genes responsible for maintenance of podocyte structure and functions with sporadic SRNS helped in identifying a distinct subgroup of SRNS [3, 4]. This group of children are generally unresponsive to immunosuppressive medications, and the disease does not recur post-transplantation [5, 6]. Thus, mutational analysis in SRNS is important because it helps in preventing unnecessary exposure to immunosuppressants and their adverse effects, besides establishing a molecular diagnosis and clear prognosis. WT1 mutations are associated with a spectrum of renal and extra-renal manifestations. Mutations in *WT1* (Wilm’s tumour 1) are detected in 5–9% of children with SRNS with a higher frequency in those with congenital or infantile onset of nephrotic syndrome and in children with Diffuse Mesangial Sclerosis [7–9]. Most of the *WT1* mutations occur mainly in exon 8 and 9, which code for zinc finger domains 2 and 3, respectively which can lead to isolated SRNS, two distinct clinical syndromes that are associated with SRNS (1) Denys-Drash syndrome (DDS) (2) Frasier syndrome (FS), and conditions without Nephrotic syndrome such as Wilm’s tumor, Meacham syndrome and somatic Mesothelioma (Online Mendelian Inheritance in Man, http://www.ncbi.nlm.nih.gov/omim database). DDS, mostly caused by mutations in exon 8 or 9 of WT1 is characterized by congenital/infantile NS, ambiguous genitalia, and a high risk for Wilms tumor while FS caused by mutations in the donor splice site at intron 9 is characterized by SRNS due to focal segmental glomerulosclerosis (FSGS), gonoadoblastoma and 46 XY disorder in sex development with sex reversal [10–12]. The current approach used for identifying pathogenic variants in the gene is Sanger’s (Direct) sequencing. It requires additional steps after amplification of the target by polymerase chain reaction (PCR) leading to a higher turnaround time. Sequencing also adds to the cost, a major constraint in emerging countries in the management of this disease. Since incidence of *WT1* mutation in SRNS is lower in Asian population, as compared to western countries, we expect that majority of the patients tested in the Indian population will have normal (wild type) sequence [13–17]. Hence a fast and cost effective high throughput screening assay to identify individuals with normal sequence is an unmet clinical need. The proposed two – step screening method will help in reducing the sequencing burden by elimination of samples with normal sequence and proceeding for Sanger’s sequencing of only those samples suspected of carrying a mutation. The approach will also aid in quickening the clinical decision making process.

One novel technique that is increasingly being used as screening tool for identification of normal or abnormal sequence pattern in the entire amplicon is the high resolution melting (HRM) analysis [18]. The principle of HRM is that a single base change in the amplicon influences the thermodynamic stability of the duplex resulting in a slight change in the melting temperature (Tm) and the fluorescence absorbance behaviour during the melting of the DNA double strand to single strands. HRM is a rapid, closed tube high throughput system wherein PCR amplification and subsequent analysis are sequentially performed in the same tube and therefore more convenient and less labor intense compared to other mutation scanning methods such as denaturing high performance liquid chromatography (DHPLC) and fluorescent multiplexed-PCR analysis (FMPA). Being a closed tube method, the risk of contamination is low. In addition, the sample identified to have a probable mutation using HRM can be further processed for sequencing thereby avoiding additional PCR. HRM also has better sensitivity and specificity than DHPLC [19]. Besides, HRM is not capital intensive since it can be performed directly with optimized primers without any other probe on a PCR instrument that collect fluorescent data with fine temperature resolution at the end of the PCR.

The aim of our study is to report for the first time the frequency and spectrum of *WT1* mutations in Indian children with sporadic SRNS from a single center. Since we observed low prevalence of pathogenic variants in exons 8 and 9 of *WT1*, we developed and validated a simple assay based on high-resolution melting analysis to quickly identify normal sequence profiles of *WT1* exon 8 and 9 in a clinical setting to exclude them from being further processed for Sanger sequencing.

## Methods

### Subjects

One hundred children diagnosed with initial SRNS or congenital nephrotic syndrome (NS) as defined by standard guidelines were included after informed consent and clinical data were recorded [20]. Children with secondary nephrotic syndrome or those who had mutation in Podocin (*NPHS2*) gene were excluded. Blood samples for mutational analysis were obtained and genomic DNA was extracted from peripheral blood leukocytes by the phenol chloroform method [21]. The quantity and quality of DNA was estimated using Nano-Drop ND-1000 (Thermo Scientific, India). To differentiate the mutations from polymorphisms, DNA from 50 healthy young adults, were used as controls.

### Mutation analysis by Sanger sequencing

Exons 8 and 9 including the splice site at intron 9 of *WT1* gene of all the recruited subjects were amplified using oligos (*WT1* _E8_F_A and *WT1* _E8_R_D for exon 8; *WT1* _E9_F_A and *WT1* _E9_R_D for exon 9) located on the intron-exon boundaries (Table 1). Oligos were designed Primer 3 plus software [22]. Bi-directional sequencing of the amplified products was performed using Big Dye Terminator v3.1 Cycle Sequencing Kit and analysed on an ABI 3730xl genetic analyser (Applied Biosystems, Foster City, CA) (Eurofins Genomics India Pvt Ltd, Bangalore, India). Sequences were evaluated for variants using the FinchTV 1.4.0 (Geospiza, Inc.; Seattle, WA, USA; http://www.geospiza.com) and NCBI BLAST.

**Table 1:** Mutagenic oligos (Round 1 PCR) used for site directed mutagenesis in exon 8 and 9 of the *WT1* gene

Mutation	Oligo Name	Oligo sequence 5’–3’	Annealing temperature (°C)
Exon 8 c.1079G > A;C360Y (hg19 - c.1283G > A; C428Y)	*WT1*_E8_F _A **E8_C428Y_R_C**	TTCCCCAAGGTGAGAAACCA ACCTTCGTTCATAGTCCTTGAAG	56
	**E8_C428Y_F_ B** *WT1*_E8_R_D	CTTCAAGGACTATGAACGAAGGT GCTGCCAGCAATGAGAAGTG	58
Exon 8 c.1119C > A;H373Q (hg19 - c.1323C > A;H441Q)	*WT1*_E8_F _A **E8_H441Q_R_C**	TTCCCCAAGGTGAGAAACCA TGTCTCCTTTGTTGTCTTTTGAG	58
	**E8_ H441Q _F_ B** *WT1*_E8_R_D	CTCAAAAGACAACAAAGGAGACA GCTGCCAGCAATGAGAAGTG	58
Exon 9 c.1180C > T;R394W (hg19 - c.1384C > T;R462W)	*WT1*_E9_F_A **E9_R462W_R_C**	TTGTTAGGGCCGAGGCTAGA TGGTCGGACCAGGAGAAC	56
	**E9_R462W_F _B** *WT1*_E9_R _D	GTTCTCCTGGTCCGACCA AGTGCGTAAACTTTTCTTCACAT	60
Exon 9 c.1190A > C;H397P (hg19 - c.1394A > C; H465P)	*WT1*_E9_F_A **E9_ H465P_R_C**	TTGTTAGGGCCGAGGCTAGA GTCTTCAGGGGGTCGGACC	60
	**E9_ H465_F_B** *WT1*_E9_R _D	GGTCCGACCCCCTGAAGAC AGTGCGTAAACTTTTCTTCACAT	52
Exon 9 c.A1200C > T;H401Y (hg19 - c.1405C > T;H469Y)	*WT1*_E9_F_A **E9_ H469Y_R_C**	TTGTTAGGGCCGAGGCTAGA GTCCTGGTGTGAGTCTTCAGGTG	58
	**E9_ H469Y_F_B** *WT1*_E9_R _D	CACCTGAAGACTCACACCAGGAC AGTGCGTAAACTTTTCTTCACAT	56

Oligos in bold indicate mutagenic oligos

### Construction of mutated sequence by site directed mutagenesis

We used the site directed mutagenesis technique to introduce a mutation in each of the exons 8 and 9 in order to be able to validate the HRM technique as described by Heish et al., 2013 [23]. The following single nucleotide change from the wild type were introduced: c.1079G > A;p.C360Y (hg19 - c.1283G > A;p.C428Y) and c.1119C > A;p.H373Q (hg19 - c.1323C > A; p.H441Q) in Exon 8 and c.1180C > T;p.R394W (hg19 - c,1384C > T;p.R462W), c.1190A > C;p.H397P (hg19 - c.1394A > C; p.H465P) and c.A1200C > T;p.H401Y (hg19 - c.1405C > T;p.H469Y) in exon 9 [24–27]. Round 1 PCR was carried out using mutagenic oligos (2 oligos per mutation; Table 1). In the second round of PCR, both the first round amplicon products were mixed in 1:1 ratio and re-amplified to produce the full length PCR products of exon 8 and 9 of the *WT1* gene using the same oligos that were used for Sanger sequencing. The insertion of point mutations was verified by Sanger sequencing.

### High resolution melt analysis for *WT1* exon scanning

The oligos sequence that was used for Sanger sequencing was also used for HRM. Standardization of the assay to determine the optimal annealing temperature and characterize the melt curve profiles of exons 8 and 9 of *WT1* gene was performed on five healthy control samples that were previously sequenced and confirmed to have wild type sequence. The standardization runs for each exon was initially performed separately and subsequently, the conditions were standardized to amplify both the exons in the same run. In brief, each reaction was performed in a final volume of 10 μl containing 20 ng of DNA, 250nM of each oligo (forward or reverse) and 1 × Melt Doctor Master mix (Thermofisher Scientific, USA). The PCR reaction conditions were as follows: initial denaturation step at 95 °C for 10 min followed by 45 cycles of 95 °C for 10s, 56 °C for 15 s, and 72 °C for 15 s. All HRM runs were performed in duplicate on Step one plus instrument (Thermofisher Scientific, USA).

For the melt analysis, the PCR products obtained after amplification were first heated to 95 °C for 1 min and then rapidly cooled to 40 °C at 1.6 °C per second to allow heteroduplex formation. The PCR products were then reheated to 95 °C at 0.01 °C per second. Melt curve was monitored (by fluorescence emission) from 40 °C to 95 °C. The melt curve analysis was performed by the resolution melt (HRM) software version 2.0 (Thermofisher Scientific, USA) using default setting. Briefly, the melt curve data for the amplicons are normalized between 0 and 100% fluorescent intensity and temperature shifted (to eliminate temperature difference between samples) for comparisons. The normalized, temperature-adjusted plot allows visual comparison of melting curve shapes to detect heterozygous variants. For the difference plot, one melting curve is chosen as a reference, and the difference between each curve and the reference is plotted against temperature to give a “fluorescence difference”. The original reference curve becomes a horizontal line at zero. Significant differences in fluorescence from the horizontal baseline indicate variations in the dissociation pattern of the amplicons. Differences are judged as significant if the technical duplicate fall outside the range. The derivative plot allowed direct visualization of the melting temperatures (Tm) and was used to differentiate between normal sequences of exon 8 and 9. To test for reproducibility, a single analyst prepared samples in duplicate and performed the HRM assay and the same analyst repeated the procedure on a different day. In addition, the melt curve prediction for both exons was carried out by the POLAND software using the nearest neighbor thermodynamic modeling, and the Blake and Delcourt algorithm [28].

For validation of the assay, five samples with mutations that were generated by site directed mutagenesis and verified by sequencing and 3 samples from healthy controls for each exon were used as positive and negative controls respectively along with three randomly selected patient samples. To further test the clinical applicability of the assay, we included additional 91 previously sequenced samples from the cohort for HRM testing and a blinded assessment was performed. The healthy control samples used in standardization and validation experiment for exon 8 and 9 were used as reference samples and the melting profiles of the amplicons were compared with that of reference samples for each exon.

## Results

### Clinical profile and *WT1* mutations in the cohort

One hundred children with sporadic SRNS (50 females, 50 males) were included in the study (see Table 2 for clinical profile). Majority of the patients were from the southern region of India (90%). The median age at the onset of nephrotic syndrome was 2.5 years (IQR 1.2–6.2 years). Parental consanguinity was found in 16% of the families. There were no children with DDS or FS. Among the patients who underwent renal biopsy, focal segmental glomerulosclerosis, minimal change disease and mesangial hyper cellularity was seen in 37, 21 and 12 patients respectively. None of the children biopsied had diffuse mesangial sclerosis (DMS). Most children received multiple immunosuppressants with variable response. Among the children who were recorded to have received calcineurin inhibitors (Cyclosporine and/or Tacrolimus), three had complete response, 11 had partial response and 40 children were calcineurin resistant. Of the 76 children with follow up data, 50 children maintained normal renal function (three complete remission, 47 had persistent proteinuria), 17 developed CKD stages II – IV, nine progressed to CKD stage V or end stage renal disease (6 received renal transplant and 3 died). The median duration for progression to CKD stage V from onset of disease was 2.7 years. Genomic DNA of all these samples were screened for mutation in exon 8 and 9 of *WT1* gene by Sanger sequencing. The samples did not show any mutation or single nucleotide polymorphism (SNP) in *WT1* gene.

**Table 2:** Clinical profile of the cohort

Characteristic	
Patients (*n*)	100 (50females/50 males)
Ethnic background (Southern region/Northern region/Eastern region; %)	90/9/1
Consanguinity (%)	12
Onset of NS (congenital/infantile/childhood primary SRNS/Unknown; %)	4/4/80/12
Age at diagnosis^a^ (years; Median, IQR)	2.5 (1.2–6.2)
Edema	74%
Proteinuria (yes/unavailable; %)	81/19
Hematuria (Yes/No/unavailable; %)	13/52/35
Hypertension (yes/no/unavailable)	35/45/20
Serum albumin (g/l; Median, Range)	1.7 (1.2–2.3)
Histopathology subtype (%)	
Minimal change disease	37
Focal segmental glomerulosclerosis	21
Mesangial hypercellularity	12
Others	9
Not performed	21
Renal outcome (%)	
Remission	4
Persistent relapse	62
Chronic Kidney disease Stage II-IV	22
End-stage renal disease	12

^a^incomplete data on 11/100 patients

### Standardization and validation of HRM assay for identifying normal sequences

**Fig. 1:**
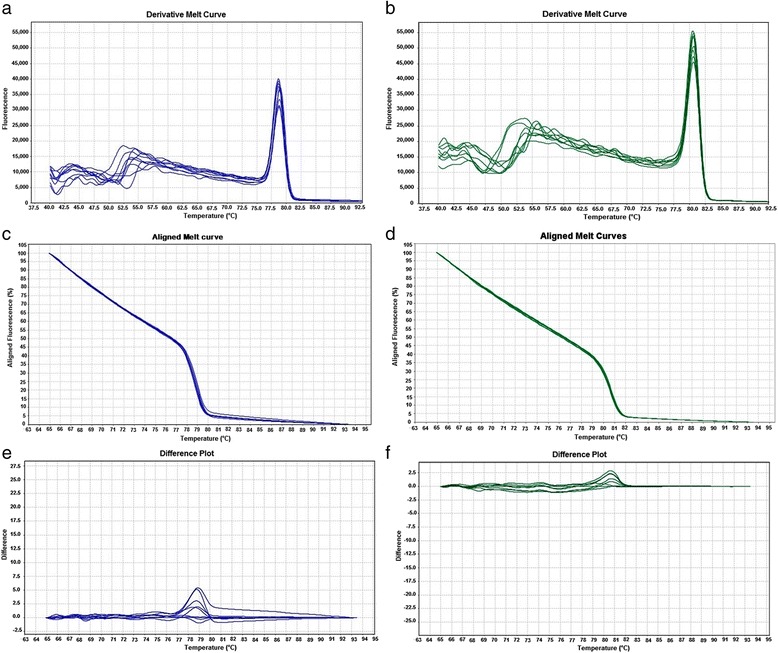
HRM data for exon 8 and 9 of *WT1* gene (standardization assay). The melt plots show five wild type control samples, which have been previously sequenced exons 8 and 9 . For each exon the Derivative plot (dF/dT) (**a**, **b**), Normalized melt curve (**c**, **d**) and Fluorescence difference plots (Δ Threshold/Temperature) (**e**, **f**) is presented

**Fig. 2:**
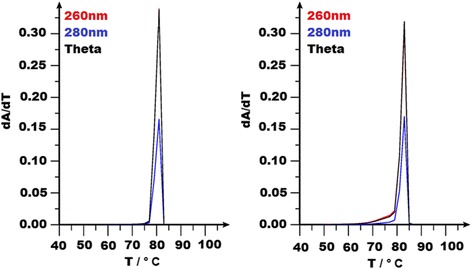
POLAND Melt profile prediction (Blake and Delcourt algorithm) for *WT1*- Exon 8 and 9. The differentiated hypochromicity at 260 and 280 nm (dA/dT) vs. temperature plot is depicted. Theta is the calculated fraction of base pairs remaining in helical state. Both exon 8 and 9 were predicted to melt completely at a single temperature and was in concordance with the experimental melt profiles

The derivative curves, aligned melt curves and the difference plots of *WT1* exon 8 and 9 of 5 healthy control samples used in the standardization run are presented in Fig. 1. Exon 8 and 9 produced unique melt curves and were visually discriminated from each other and they could be detected in the same PCR run. The melting temperature (standard deviation) of exon 8 and 9 were 78.8 °C (±0.15) and 80.5 (±0.1) respectively. We observed high reproducibility of the melting temperature (Tm) values for a given exon. The melt profiles of *WT1* exon 8 and 9 obtained in the standardization run was in concordance with the melt profile obtained using the nearest neighbor thermodynamic modeling and the Blake and Delcourt algorithm (POLAND software) (Fig. 2).

**Fig. 3:**
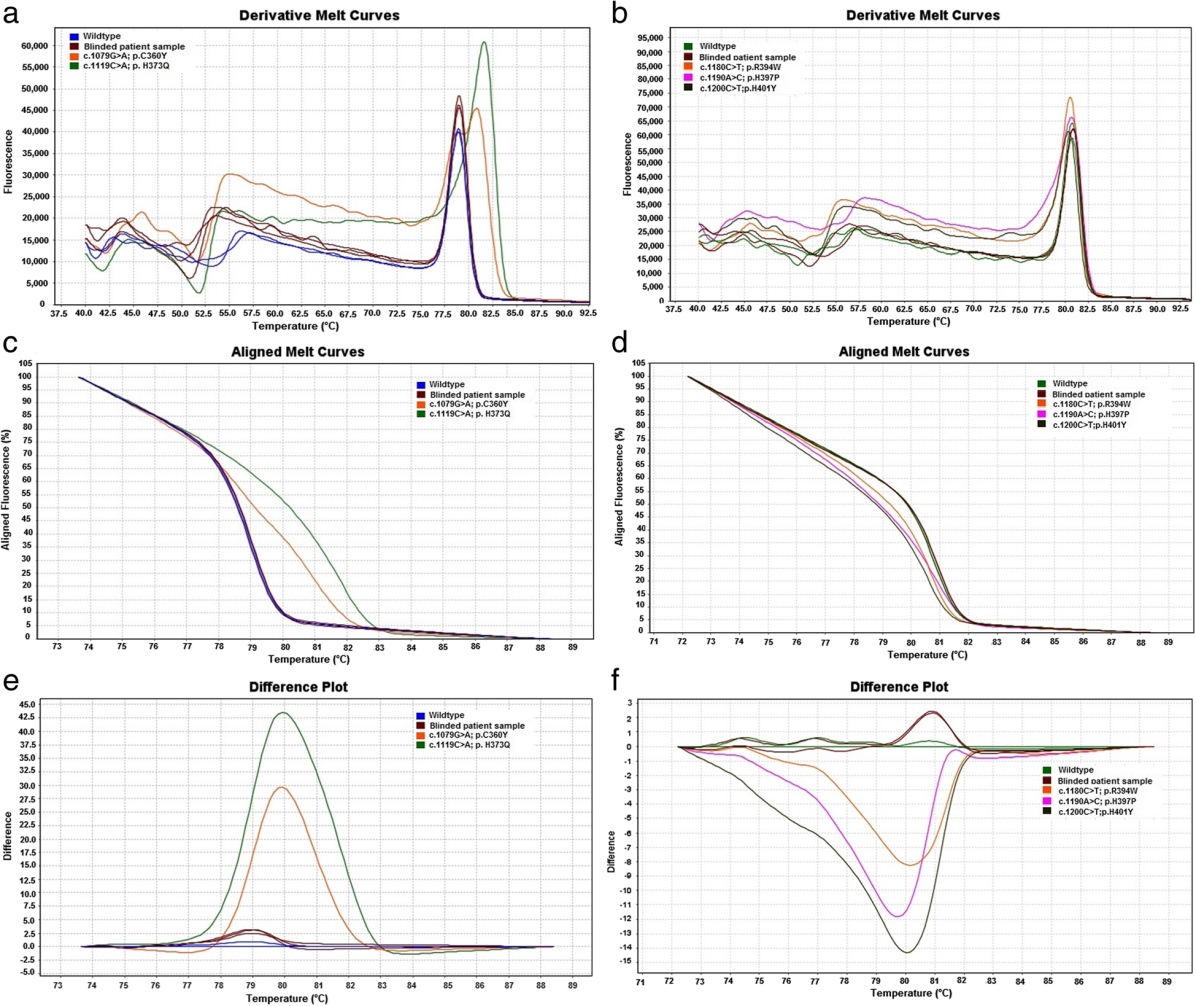
HRM Validation assay showing derivative melt plot, normalized melt curve and difference plots for exon 8 and 9 of the *WT1* gene. In *WT1* exon 8, the wildtype (*blue*) and the blinded patient sample (*maroon*) grouped together indicating that they have the same genotype. The mutant samples c.1079G > A; p.C360Y (*orange*) and c.1119C > A; p.H373Q (*green*) were distinguished from the wildtype sample (Fig. 3 **a**, **c** and **e**). Similary in *WT1*exon 9, the wildtype (*green*) and the blinded patient sample (*maroon*) grouped together and the mutant samples c.1180C > T; p.R394W (*red*), c.1190A > C; p.H397P (*pink*) and c.A1200C > T; p.H401Y (*black*) were easily distinguished from the wild type (Fig. 3 **b**, **d** and **f**)

The method was validated using the five positive mutated sequences obtained by site directed mutagenesis methodology along with three healthy controls (wild type) and three randomly chosen patient samples. All the samples with mutation had a distinctive melting pattern which was visually discriminated when compared with those of the wild-type sequences indicating the ability of the HRM assay to discriminate mutated from wild type samples (Fig. 3). The HRM profile of patient samples was similar to that of healthy controls which were verified by sequencing.

To further test the clinical application of this assay, we screened 91 patient samples that had been previously sequenced for Exon 8 and 9 of *WT1* gene with the healthy control samples as reference samples. The analysis of the HRM data was done independently by two observers in the following way: the curves were first auto grouped by the software based on the melt transition. The observers, who were blinded to the identity of the samples, confirmed that the Tm difference obtained between the exon 8 and 9 was same as that obtained in the standardization assay. The heterozygotes if any were to be identified by the shapes of their melting curves. There was no change in the classification by the observers as compared to that grouped by the software. The Tm of the amplicons, exon 8 and 9 were unambiguously distinguished from each other in the derivative plot (Fig. 1a). The two exons were also readily distinguishable in the aligned melting curve and in the difference plots (amplitude and/or shape of the curves: Fig. 1b and c). Exons 8 and 9 of all the patient samples had similar melt profiles as that of their respective reference sample and reliably sorted into one of the two distinct groups (Additional file 1: Figure S1). The melt profile results were identical to the Sanger’s sequencing results. The HRM results were obtained in 2 h 15 min, while sequencing required a minimum of 5 days to obtain the final result. The cost analysis calculated in the present conditions showed that conventional PCR followed by Sanger sequencing was three times costly when compared to high-resolution melting assay (Additional file 2: Table S1).

## Discussion

SRNS is a genetically heterogeneous disorder with a broad phenotypic spectrum [29, 30]. Our previous study indicated that the prevalence of Podocin mutation in Indian children with SRNS was low as compared to the Western countries [31]. The present study from a single centre in India comprising largely of patients from the southern region of the country (90%) was conducted to determine whether *WT1* mutations could be the cause of sporadic SRNS in a subset of patients who did not have Podocin mutation. In our cohort, no subject tested positive for mutation in *WT1*. Based on the number of children tested, the prevalence of WT-1 mutation in Indian children with SRNS with 95% confidence is ≤ 3% [32]. The prevalence of *WT1* mutation in children with SRNS varies according to the population origin and is summarised in Table 3. Data from large cohort studies that included children with SRNS from various ethnic background like central and west European, Turkish, American, African-American, Hispanic, South Asian, Arabic, or African, shows that the prevalence of *WT1* mutation varies between 1.7 and 9% [9, 26, 33]. Studies limited to specific ethnic groups also show wide variation in the prevalence of *WT1* mutation with low prevalence in Japanese and Chinese population and high prevalence in Greek, Spanish and Korean population [13–16, 34, 35]. The location and type of WT1 mutations can predict clinical features, histology and renal outcomes. In the largest cohort study of 61 children with WT1 mutations, Lipska et al. observed that while missense mutations were associated with DMS, early onset SRNS and rapid progression to ESRD, the truncating mutations were seen in those who had late onset of SRNS and these children were also at high risk for Wilm’s tumor [9]. In the same study isolated SRNS was associated with intronic (KTS) mutations and slow progression to ESRD. A similar observation was also reported by Chernin et. al., who observed that intronic mutations (KTS) cause NS with slower progression to ESRD compared with missense mutation [36]. In another retrospective analysis of 53 mid-European patients, those with missense mutations required dialysis significantly earlier that those with truncating mutations [37]. We could not perform genotype-phenotype correlation due to absence of mutations in our study. The average age of onset of nephrotic syndrome in our cohort is very similar to that reported in other studies suggesting that age cannot explain the low prevalence *WT1* mutation in our cohort [14–17, 26, 33, 34, 38]. As shown in Table 3 the presence of syndromic SRNS (DDS or FS or patient with genitourinary tract malformations) can also influence the frequency *WT1* mutation. In the study by Mucha et al. the prevalence of *WT1* mutation decreased from 6. 3 to 3.9% when patients with genitourinary tract malformations were excluded [33]. Being a single centre study with most of the patients from the southern region, our findings cannot to be generalized to the Indian population. The present cohort comprised of children with only isolated SRNS and none of the patients had DMS on biopsy. The probability of detecting mutations in WT 1 are higher in children with DDS and FS or in those with DMS. In addition, we screened only the hot spot regions of exon 8 and 9 that account for majority of mutations and not the complete open reading frame of WT1 gene. These reasons could potentially explain the absence of *WT1* mutation observed.

**Table 3:** Detection rates of *WT1* mutations in children with SRNS in different ethnic groups

Ethnic background	Total (Female/Male)	Age of Onset (Median; Years)	Number of cases with *WT1* mutation (%)	Prevalence of *WT1* mutation in isolated SRNS (%)
Korean [35, 43]	424 (39/31)	4.7	25 (5.9%)	8 (1.9%)
Japanese [44]	13 (10/3)	0–1 month	0	0
Chinese [14]	52 (14/38)	3.5	1 (1.9%)	1 (1.9%)
Italian [17]	64 (32/32)	6.8	4 (6.2%)	2 (3%)
Spanish [45]	125 (NA)	1.3	5 (4%)	NA
Greek [16]	27 (17/10)	8	4 (14.8%)	3 (11%)
Europe and Middle East [9]	761	2.0	61 (8%)	17 (2.23%)
Worldwide cohort^a^ [33]	167 (80/87)	5.5	15 (9%)	11 (6.6%)
^b^Worldwide [7]	2016 (943/1067)	3.4	35 (1.7%)	32 (1.6%)
Indian (Current study)	100 (50/50)	2.6	0 (0%)	0 (0%)

*NA* not available

^a^Central European, Turkish, African-American, Hispanic, or Asian

^b^Central Europe, Turkey, and India

The heterogenic nature of SRNS makes mutation analysis costly and time consuming. Since the prevalence of the *WT1* mutation is observed to be low, we standardized a simple and economical assay that will be useful in the clinic to identify quickly and accurately subjects with no mutation. We chose HRM assay as a screening tool because it has been found to be a very useful high throughput mutation scanning method in various human diseases [39–41]. The oligo designed for Sanger sequencing were also suitable for HRM analysis, and hence there was no need to design separate set of oligos. Further, the PCR conditions were optimized in such way that both exons can be tested in a single run. Performing simultaneous analysis of the two exons in the same run saves time as well as cost. The melting curves generated in this study allowed unambiguous differentiation of exon 8 and 9 and were highly reproducible. This is the first report wherein a single HRM run can be used for screening of both exons 8 and 9 of *WT1* gene. Our HRM results were in complete concordance with conventional Sanger sequencing suggesting that the HRM gene scanning method can be used to screen for *WT1* gene mutation. In contrast to Sanger sequencing, the use of HRM allows to considerably lower the costs of gene mutation screening in the clinic by reducing the number of samples subjected to Sanger sequencing. Use of this method could reduce costs to 9$ per sample when compared to Sanger sequencing which is 30$ per sample, thus proving to be a cost effective method for gene scanning. We also noted that the turnaround time is much lower with HRM. The entire analysis, including the preparation of the reaction mixture, amplification and melting of the products takes a little more than 2 h and interpretation of the result takes 30 min (Additional file 2: Table S1). Further, HRM analysis require less manpower as single technician can perform the test and screen large number of samples in a single assay. Since the assay only requires real time PCR equipment, clinical laboratories can initiate similar screening strategy using our standardized protocol. The same technique could be used in screening other genes and risk alleles associated with SRNS in a similar way. The *APOL1* G1 and G2 risk alleles in the African American population with FSGS is significantly enriched as compared to the general population. HRM will be an ideal technique to rapidly screen for these alleles in our population [42].

In summary, no pathogenic variant in exon 8 and 9 of *WT1* gene was found in the present cohort. We developed a simple, high throughput approach for identification of normal variants in *WT1* gene which is six times faster and three times less cost compared with traditional direct sequencing. The results obtained by HRM on the clinical samples further support the feasibility of applying the assay as a screening technique in a clinical setting.

## Additional files

Additional file 1: Figure S1. HRM analysis of cohort for exon 8 and 9 of *WT1* gene. The melt plots show wild type control samples and representative patient samples, which have been previously sequenced exons 8 and 9 . For each exon the Derivative plot (dF/dT) (A,B), Normalized melt curve (C, D) and Fluorescence difference plots (Δ Threshold/Temperature) (E, F) is presented. (TIF 3577 kb)
Additional file 2: Table S1. Summary of the properties of High Resolution Melt and Direct sequencing. (DOCX 12 kb)

## Abbreviations

CKD: Chronic kidney disease
DDS: Denys-Drash syndrome
DHPLC: Denaturing high performance liquid chromatography
DMS: Diffuse mesangial sclerosis
ESRD: End-stage renal disease
FMPA: Fluorescent multiplexed-PCR analysis
FS: Frasier syndrome
HRM: High resolution melt
NS: Nephrotic syndrome
PCR: Polymerase chain reaction
SNP: Single nucleotide polymorphism
SRNS: Steroid resistant nephrotic syndrome
Tm: Melting temperature
*WT1*: Wilm’s tumor 1
