# Supplementary figures and images for: Screening of WT1 mutations in exon 8 and 9 in children with steroid resistant nephrotic syndrome from a single centre and establishment of a rapid screening assay using high-resolution melting analysis in a clinical setting
Source: BMC Med Genet. 2017 Jan 10;18:3. doi: 10.1186/s12881-016-0362-7 (PMC5223455; doi:10.1186/s12881-016-0362-7)

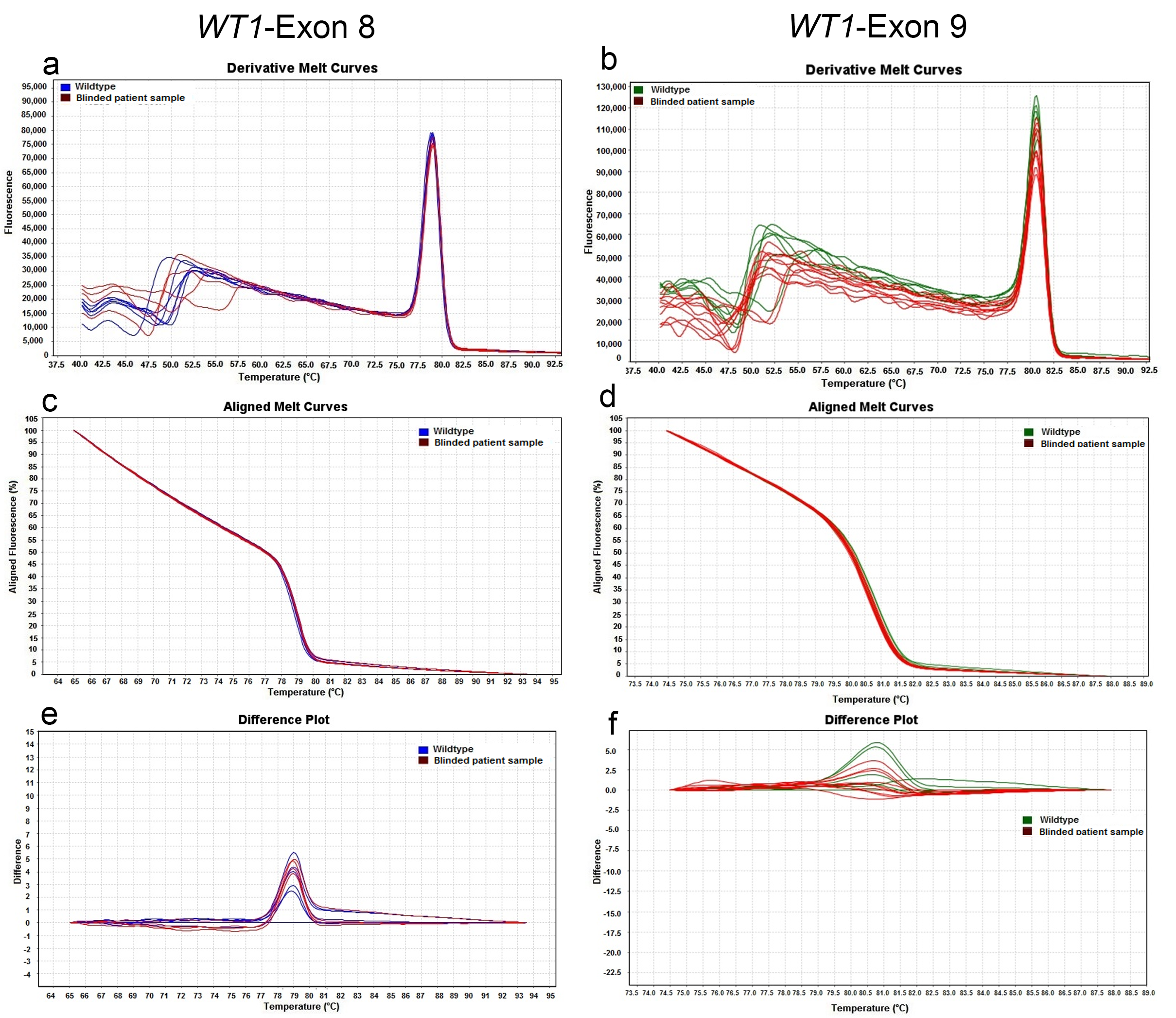

Supplement: Additional file 1: Figure S1. — HRM analysis of cohort for exon 8 and 9 of WT1 gene. The melt plots show wild type control samples and representative patient samples, which have been previously sequenced exons 8 and 9 . For each exon the Derivative plot (dF/dT) (A,B), Normalized melt curve (C, D) and Fluorescence difference plots (Δ Threshold/Temperature) (E, F) is presented. (TIF 3577 kb) [file 12881_2016_362_MOESM1_ESM.tif]
