# Supplementary material for: Screening of WT1 mutations in exon 8 and 9 in children with steroid resistant nephrotic syndrome from a single centre and establishment of a rapid screening assay using high-resolution melting analysis in a clinical setting
Source: BMC Med Genet. 2017 Jan 10;18:3. doi: 10.1186/s12881-016-0362-7 (PMC5223455; doi:10.1186/s12881-016-0362-7)
Supplement: Additional file 2: Table S1. — Summary of the properties of High Resolution Melt and Direct sequencing. (DOCX 12 kb) [file 12881_2016_362_MOESM2_ESM.docx]

| **Parameters** | **High Resolution Melt** | **Direct Sequencing** |
| --- | --- | --- |
| Amount of input DNA | 20ng/reaction | 50ng/reaction |
| Technician time | 5 hours | 7 hours |
| Time for analysis | 30 minutes | 3-4 hours |
| Turnaround time | ½ day | 2-3 days |
| Ease of interpretation | medium | easy |
| Cost |  |  |
| Reagent cost | 2.38 $ | 15.28$ |
| Instrument charges | Real time PCR cycler- 4.08$ | PCR-3.77 $ Sequencing* -10.74 $ |
| Man hour charges | 2.5$ | * |
| Total | 8.96 $ | 29.79 $ |
| ^*outsourced^ |  |  |
